# An Autoencoder and Machine Learning Model to Predict Suicidal Ideation with Brain Structural Imaging

**DOI:** 10.3390/jcm9030658

**Published:** 2020-02-29

**Authors:** Jun-Cheng Weng, Tung-Yeh Lin, Yuan-Hsiung Tsai, Man Teng Cheok, Yi-Peng Eve Chang, Vincent Chin-Hung Chen

**Affiliations:** 1 Department of Medical Imaging and Radiological Sciences, Chang Gung University, Taoyuan 33302, Taiwan; jcweng@mail.cgu.edu.tw (J.-C.W.); zlink0587@gmail.com (T.-Y.L.); mantengcheok0723@gmail.com (M.T.C.); 2 Medical Imaging Research Center, Institute for Radiological Research, Chang Gung University and Chang Gung Memorial Hospital at Linkou, Taoyuan 33302, Taiwan; 3 Department of Psychiatry, Chang Gung Memorial Hospital, Chiayi 61363, Taiwan; 4 School of Medicine, Chang Gung University, No. 259, Wenhua 1st Rd., Guishan Dist., Taoyuan 33302, Taiwan; russell.tsai@gmail.com; 5 Department of Diagnostic Radiology, Chang Gung Memorial Hospital, Chiayi 61363, Taiwan; 6 Department of Counseling and Clinical Psychology, Columbia University, New York City, NY 10027, USA; tiramisueve@gmail.com

**Keywords:** suicidal ideation, autoencoder, machine learning, generalized q-sampling imaging

## Abstract

It is estimated that at least one million people die by suicide every year, showing the importance of suicide prevention and detection. In this study, an autoencoder and machine learning model was employed to predict people with suicidal ideation based on their structural brain imaging. The subjects in our generalized q-sampling imaging (GQI) dataset consisted of three groups: 41 depressive patients with suicidal ideation (SI), 54 depressive patients without suicidal thoughts (NS), and 58 healthy controls (HC). In the GQI dataset, indices of generalized fractional anisotropy (GFA), isotropic values of the orientation distribution function (ISO), and normalized quantitative anisotropy (NQA) were separately trained in different machine learning models. A convolutional neural network (CNN)-based autoencoder model, the supervised machine learning algorithm extreme gradient boosting (XGB), and logistic regression (LR) were used to discriminate SI subjects from NS and HC subjects. After five-fold cross validation, separate data were tested to obtain the accuracy, sensitivity, specificity, and area under the curve of each result. Our results showed that the best pattern of structure across multiple brain locations can classify suicidal ideates from NS and HC with a prediction accuracy of 85%, a specificity of 100% and a sensitivity of 75%. The algorithms developed here might provide an objective tool to help identify suicidal ideation risk among depressed patients alongside clinical assessment.

## 1. Introduction

Suicide is an important and serious worldwide public health problem. In 2012, approximately 800,000 people died from self-inflicted injuries [1]. Suicide is conceptualized as a continuum of ideations/behaviors, including suicidal ideation, suicide planning, attempted suicide, and completed suicide [2]. An international survey showed a 9.2% prevalence of lifetime suicidal ideation [3]. Only 0.8–10% of suicide attempters reported the absence of previous suicidal ideation [2]. Overall, suicidal ideation emerges as the first stage of the suicide process and a potential gate for suicide prevention.

Suicide risk evaluation still relies on clinicians’ experiences, and many health-related workers are not familiar with suicide risk assessment. However, patients’ denials of suicidal ideation can lower the vigilance of clinicians’ assessments, even in persons with a known suicide risk [2]. Very high rates of suicide among post-discharge patients reflect the limitations of assessments of suicide risk based on existing strategies. [4]. Monitoring suicidal ideation behavior should therefore not rely heavily on such denials [2]. Thus, it is important to create new objective methods to evaluate the risk of suicide.

Recently, neuroimaging studies have shown that suicidal behavior is a state-based condition stored in the neural circuitry that can be quickly switched on by the recall of an experience of mental pain [5]. Myung et al. used diffusion tensor imaging (DTI) to assess topological organization changes of white matter networks according to suicidal ideation in major depressive disorder (MDD) patients. They suggested that the reduced structural connectivity of the frontosubcortical circuit, including regions associated with executive function and impulsivity, is important in the emergence of suicidal ideation among MDD patients [6]. Taylor et al. used anatomical analyses and DTI and found that a depressed group with suicidal ideation exhibited reduced cortical thickness in the frontoparietal regions and the insula. Depressed patients with suicidal ideation also showed widespread white matter differences in fractional anisotropy and radial diffusivity. These differences were observed primarily in posterior parietal white matter regions and central white matter tracts adjacent to the basal ganglia and thalamus [7].

Traditional statistics can only differentiate different groups with suicidal ideation from those without, rather than detect which individual is at risk. New analytical methods, such as machine learning, have been used in an attempt to develop algorithms to classify individual risk [8,9,10,11]. One study used machine learning algorithms based on functional magnetic resonance imaging (fMRI) neural signatures of death- and life-related concepts to detect individuals with suicidal ideation with 91% accuracy [12]. There have been few related studies exploring whether machine learning methods of structural magnetic resonance imaging (MRI) can be additional tools to identify patients with suicidal ideation.

The present study aimed to investigate whether MRI-measured structural changes can assist in suicide risk stratification among individuals with suicidal ideation and non-suicidal ideation populations (depressed patients and healthy controls) using machine learning-based analysis.

## 2. Methods

### 2.1. Participants

In our study, we recruited 153 participants which were in one three groups of: 41 depressive patients with suicidal ideation (SI; age 21–60 years, mean = 42.17 years, SD = 11.85 years), 54 depressive patients without suicidal thoughts (NS; age 21–60 years, mean = 46.17 years, SD = 9.86 years), and 58 healthy control subjects (HC; age 20–57 years, mean = 40.22 years, SD = 9.65 years). All participants were at least 20 years of age and right-handed. The confirmation of MDD, suicide ideation was primarily based on psychiatrists’ diagnosis and the Mini-International Neuropsychiatric Interview (MINI) carried out by the trained research nurse. The final confirmation was carried out by the principle investigator (V.C.-H.C.) according to all available information. The participants were recruited via the outpatient clinic of the department of psychiatry at Chiayi Chang Gung Hospital and recruitment advertisements. Exclusion criteria for all participants were any eye diseases (e.g., cataract and glaucoma), history of suicide attempt, another primary severe mental disorder (e.g., schizophrenia or bipolar disorder), alcohol/illicit substance use disorder during the past year, any neurological illnesses, and metallic implants or other contraindications for MRI. The study was approved by the Institutional Review Board of Chang Gung Memorial Hospital, Chiayi, Taiwan (No. 104-0838B, 104-9337B, 201602027B0). All participants joined in the study after providing informed consent, and all research was performed in accordance with relevant guidelines and regulations.

### 2.2. Diffusion Magnetic Resonance Imaging Data Acquisition

All participants were scanned using a 3 T MRI (Verio, SIEMENS, Munich, Germany) system with a single-shot diffusion-weighted spin echo-planar imaging sequence. Diffusion images were obtained with repetition time (TR)/ echo time (TE) = 8943/115 ms, field of view (FOV) = 250 × 250 mm^2^, matrix = 128 × 128, slices = 35, in-plane resolution = 2 × 2 mm^2^, slice thickness = 4 mm, signal average = 1192 noncollinear diffusion weighting gradient directions with b = 1000, 1500, 2000 s/mm^2^, and one null image without diffusion weighting (b = 0 s/mm^2^).

### 2.3. Generalized q-Sampling Imaging Analysis

Generalized q-sampling imaging (GQI) is a new reconstruction method based on the Fourier transform between the diffusion magnetic resonance (MR) signals and the diffusion displacement, which allows the deduction of a new relationship between the directly estimated spin distribution function (SDF) and the diffusion MR signals. GQI is a model-free method that quantifies the density of water, which diffuses in different orientations. Model-free methods estimate the empirical distribution of water diffusion with no hypotheses. The SDF is the density of diffusing water in different directions and is a kind of diffusion orientation distribution function (ODF). Generalized q-sampling imaging provides information on the relationship between the diffusion signals of water and the SDF; therefore, GQI can provide directional and quantitative information about crossing fibers. Studies have shown that GQI has good sensitivity and specificity for white matter properties and pathology [13].

In GQI analyses, eddy correction was first performed using FSL (FMRIB Software Library, Oxford, UK), and the corrected diffusion images were subsequently registered to the null image in native diffusion space with a linear transformation. The registered diffusion images were then mapped to the standard T2-weighted template after an affine transformation with 12 degrees of freedom and nonlinear warps using statistical parametric mapping (SPM, Wellcome Department of Cognitive Neurology, London, UK) After the preprocessing procedure, GQI index mapping was reconstructed from multi-shell diffusion data using DSI studio (National Taiwan University, Taipei, Taiwan), including generalized fractional anisotropy (GFA), normalized quantitative anisotropy (NQA), and the isotropic value of the orientation distribution function (ISO). GFA is calculated from an ODF, and GFA is defined as the standard deviation divided by the root mean square of the ODF, indicating a measurement of the anisotropy of water diffusion in microstructures. NQA is the normalized QA, which is defined as the amount of anisotropic spins that diffuse along the fiber orientation. ISO is the minimum distribution value of an ODF and thus represents the background isotropic diffusion [13].

### 2.4. Autoencoder and Supervised Machine Learning Analysis

**Figure 1:**
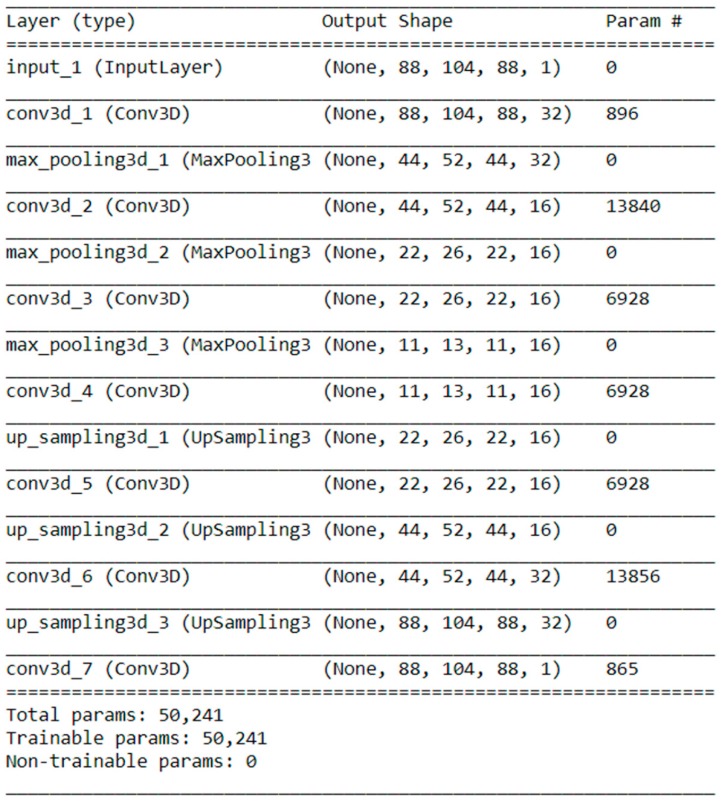
The structure of the autoencoder, which consists of six 3D convolution layers, three max-pooling layers, and three upsampling layers, resulted in compressed images with sizes of (11, 13, 11, 16) for feature extraction.

To conduct the largest number of clinically required trials possible that target ideation subjects from non-suicide-attempt subjects, we attempted to classify ideation subjects separately from the others except for those who had previously attempted suicide. In other words, this was a binary classification of ideation subjects and non-ideation subjects, the latter of which included the HCs and depression subjects. In this analysis, we employed a 3D autoencoder for feature extraction in the GQI dataset, which contained GFA, ISO, and NQA maps of the healthy controls, depressed subjects, ideation subjects, and suicidal attempt subjects (58, 54, 41, and 33 subjects, respectively). For the simplicity of convolutional neural network (CNN) design, the edges of the GQI images were pruned to change the size from (91; 109; 91) to (88; 104; 88) before being sent to the CNN-based autoencoder model. The structure of the network is shown in Figure 1. In addition to the structural MRI data, the autoencoder was compiled with an Adadelta optimizer along with a learning rate of 0.01 and a binary cross-entropy loss function in 50 epochs. Following autoencoder feature extraction, the supervised machine learning algorithm extreme gradient boosting (XGB) and logistic regression (LR) were used to discriminate ideation subjects from HCs and depression subjects using the resulting compressed images, the sizes of which were (11; 13; 11; 16) and flattened to (25,168).

Considering the imbalanced proportion of non-ideation to ideation subjects (112:41), we decided to split the non-ideation subjects into two halves. Each half contained 29 HCs and 27 depression subjects and were then concatenated with the ideation subjects and used for training and testing. In the dataset, the proportion of training set, validation set, and testing set were 4:1:1 by random selection, which means 5-fold cross validation (CV) and independent separate testing set were used. The stratify parameter in train_test_split was used to obtain a 4:1 ratio of the training set and testing set. A 10-fold iteration was performed on train_test_split to obtain results with less bias. In detail, to prevent overfitting, the data was randomly separated into training and test sets 10 times using an iteration of train-test split. The numbers, from 1–9, were assigned to the random_state parameter. The process was designed to keep the model from overfitting a single train and test set. For tuning of the XGB, the max_depth and n_estimators parameters were set to 5 and 1000, respectively, while the default values of the remaining XGB parameters and all parameters of LR were used in scikit-learn [14]. The average accuracy, sensitivity, specificity, and area under curve (AUC) of each result were recorded.

## 3. Results

### 3.1. Participants

Table 1 shows the demographic characteristics of the participants. There were significant differences in age, gender, and years of education among the three groups. Therefore, these parameters were used as covariates for subsequent analyses.

### 3.2. Autoencoder and Supervised Machine Learning Analysis

**Figure 2:**
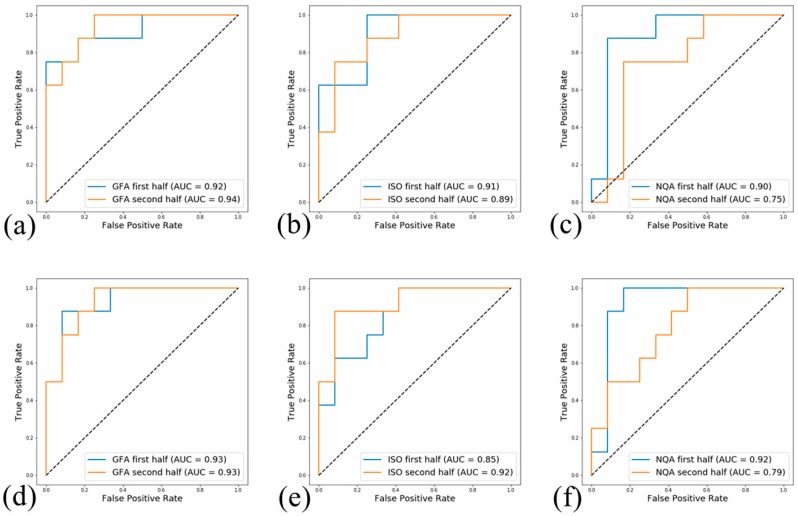
The testing set classification receiver operating characteristic (ROC) curves showing the best results of (**a**) extreme gradient boosting (XGB)- generalized fractional anisotropy (GFA), (**b**) XGB- isotropic value of the orientation distribution function (ISO), (**c**) XGB- normalized quantitative anisotropy (NQA), (**d**) logistic regression (LR)-GFA, (**e**) LR-ISO, and (**f**) LR-NQA following autoencoder and supervised machine learning analysis, along with the area under curves (AUCs) (AUC: XGB-GFA-1 = 0.92; XGB-GFA-2 = 0.94; XGB-ISO-1 = 0.91; XGB-ISO-2 = 0.89; XGB-NQA-1 = 0.90; XGB-NQA-2 = 0.75; LR-GFA-1 = 0.93; LR-GFA-2 = 0.93; LR-ISO-1 = 0.85; LR-ISO-2 = 0.92; LR-NQA-1 = 0.92; LR-NQA-2 = 0.79).

The results of the supervised machine learning binary classification, distinguishing the ideation subjects from HCs and depression subjects via LR and XGB, are shown in Table 2 and Table 3. The results show the accuracy (ACC), sensitivity (SEN), specificity (SPE), and AUC of the 5-fold CV and testing set. In Table 2, each metric is an average result of a 10-fold iteration, while the results with the highest AUC are presented in Table 3. As mentioned in the methods, the data from the HCs and depression subjects were combined and then split into halves. The two halves were separately distinguished from the ideation subjects and denoted as -1 (first half) or -2 (second half). Receiver operating characteristic (ROC) curves of the test results having maximum AUCs are depicted in Figure 2. Among the averaged results, the highest CV accuracy, 0.74, was attained via XGB-ISO-2. The highest CV AUC, 0.81, was attained via XGB-ISO-2. The highest testing accuracy, 0.73, was attained via XGB-GFA-1 and XGB-ISO-2. The highest testing AUC, 0.84, was obtained with XGB-ISO-2. Among the best results, the highest CV accuracy, 1.00, was attained via XGB-GFA-1. The highest CV AUC, 1.00, was attained via XGB-GFA-1, XGB-ISO-2, LR-GFA-2, LR-ISO-2, and LR-NQA-1. The highest testing accuracy, 0.95, was attained via XGB-GFA-1. The highest testing AUC, 0.97, was attained via XGB-ISO-2.

## 4. Discussions

To our knowledge, this is the first study using a machine learning algorithm based on the structural MRI to identify individuals with suicidal ideation. The results showed that the best pattern of structures across multiple brain locations can classify suicidal ideates from non-ideation depressed patients and healthy controls with a prediction accuracy of 85%, a specificity of 100%, and a sensitivity of 75%.

Compared to previous studies, we used a different approach to determine the effect of machine learning. One neurosemantic study used machine learning algorithms based on fMRI neural signatures of death- and life-related concepts to detect youth with suicidal ideation with 91% accuracy [12]. Based on this approach, they distinguished 17 suicidal ideates from 17 controls with no diagnosis of depression. This accuracy is similar to that from our findings. Just proposed a concept-based study to detect alterations in the neural representations of concepts related to death and life. The approach provided a profound method to explore theories about suicidal ideation. Since assessments based on task-based functional MRI require more time and higher technique requirements, our machine learning classifier based on the structural MRI provides another feasible clinical method to detect at-risk suicidal ideates.

In previous traditional structural MRI studies [6,7], the results showed reduced structural connectivity of the frontosubcortical circuit [6], reduced cortical thickness in the frontoparietal regions and the insula, and widespread white matter differences in posterior parietal white matter regions and central white matter tracts adjacent to the basal ganglia and thalamus [7]. Traditional MRI analysis can only discriminate the difference between groups with suicidal ideation from those without suicidal ideation. Newer analysis methods, such as the machine learning algorithm used in this study, can classify individual risks. This approach more closely resembles risk assessment in real-world clinical practice.

In our autoencoder and supervised machine learning analysis, we first tried to use scale_pos_weight in the XGB model, controlling the balance of positive and negative weights, to reduce the imbalance in the proportion of ideation patients to non-ideation participants. Generally, the value of this parameter should be set as the ratio of negative samples to positive samples, which was 2.73 (112/41) in our case. However, the imbalance remained strong after tuning, resulting in a high specificity and a low sensitivity (for example, GFA: CV accuracy = 0.74; testing accuracy = 0.77; CV sensitivity = 0.42; testing sensitivity = 0.49; CV specificity = 0.87; testing specificity = 0.87; CV AUC = 0.76; testing AUC = 0.77; the results were the averages of a 10-fold train_test_split iteration). Thus, we decided to adopt the current method, dividing the dataset of HCs and depression subjects into two halves.

Another issue that may exist is the high dimensionality of the imported feature set, which contained 25,168 features for each subject that remained even after 3D-CNN compression. As a result, feature selection methods were also used to reduce the dimensionality but did not yield better results. In addition to the XGB algorithm, support vector machine (SVM) and random forest (RF) were utilized for prediction as well, but inferior results were attained compared with XGB and LR.

There are some strengths in the present study. First, we used several different machine learning methods to determine the most appropriate algorithm. Second, the diagnosis of depression was based on clinical assessment, and confirmation of suicidal ideation was based on both self-report and structural interviews. Third, we recruited both nondepressed and depressed control subjects to control for the effect of depression. The sample size was large compared to the only previous study. Notwithstanding, this study had some limitations. A total of nine classification machine learning models were used in this study including: (1) logistic regression (LR); (2) XGBoost (XGB); (3) decision tree classifier (CART); (4) linear discriminant analysis (LDA); (5) Gaussian na.ve Bayes (NB); (6) k-nearest neighbors classifier (KNN); (7) support vector machine (SVM); (8) multilayer perceptron (MLP); and (9) random forest (RF) [15]. However, only XGB and LR showed significant predictions on classifying GQI images into suicidal ideation and non-ideation subjects. Since different accuracies were obtained among the different methods tested, it may imply that more samples are warranted.

## 5. Conclusions

The results from the present study showed the good accuracy of a machine learning algorithm based on data from structural MRI, and it might provide an objective tool to help identify suicidal ideation risk among depressed patients alongside clinical assessment.

## Figures and Tables

**Table 1:** Summary of characteristics of demographic data and neuropsychological tests.

Characteristics	Depressive Patients with Suicidal Ideation (SI; *n* = 41)		Depressive Patients without Suicidal Ideation (NS; *n* = 54)		Healthy Controls (HC; *n* = 58)		A (SI vs. NS)	B (SI vs. HC)	C (NS vs. HC)
	Mean	SD	Mean	SD	Mean	SD	*p*-Value		
Age	42.17	11.85	46.17	9.86	40.22	9.65	0.08	0.39	0.002
Age range	21–60	N/A	21–60	N/A	20–57	N/A	N/A	N/A	N/A
Gender (M/F)	18/23	N/A	23/31	N/A	7/51	N/A	0.90	0.0007	0.0003
Education (years)	13.17	3.36	13.19	2.85	14.11	2.94	0.98	0.585	0.092
HAM-D	17.49	6.17	14.96	6.54	4.5	5.85	0.058	<0.0001	<0.0001
Anxiety of HADS	12.05	4.09	8.00	4.53	4.75	3.61	<0.0001	<0.0001	<0.0001
Depression of HADS	12.56	4.43	7.09	4.62	3.85	3.27	<0.0001	<0.0001	<0.0001

A, T-test between depressive patients with suicidal ideation and depressive patients without suicidal thoughts; B, T-test between depressive patients with suicidal ideation and healthy controls; C, T-test between depressive patients without suicidal ideation and healthy controls; HADS, Hospital Anxiety and Depression Scale; HAM-D, Hamilton depression rating scale; N/A, not applicable; SD, Standard deviation; *p*-value < 0.05 indicating significant difference.

**Table 2:** The results of identifying ideation subjects from healthy control (HC) subjects and depression subjects by extreme gradient boosting (XGB) and logistic regression (LR) are the average of a 10-time train_test_split iteration. The data of HCs and depression subjects was split into two halves (−1 and −2) to reduce imbalance. ACC, accuracy; AUC, area under curve; SEN, sensitivity; SPE, specificity.

Model	Metric	5-Fold cross validation (CV)				Test			
		ACC	SEN	SPE	AUC	ACC	SEN	SPE	AUC
XGB	GFA-1	0.67	0.55	0.79	0.77	0.73	0.65	0.78	0.82
	GFA-2	0.61	0.53	0.69	0.66	0.64	0.55	0.71	0.72
	ISO-1	0.63	0.51	0.73	0.66	0.67	0.55	0.75	0.69
	ISO-2	**0.74**	0.65	0.81	**0.81**	**0.73**	0.68	0.78	**0.84**
	NQA-1	0.63	0.47	0.75	0.71	0.63	0.54	0.69	0.70
	NQA-2	0.60	0.49	0.69	0.64	0.59	0.41	0.71	0.61
LR	GFA-1	0.67	0.54	0.79	0.76	0.69	0.56	0.77	0.77
	GFA-2	0.65	0.56	0.74	0.75	0.68	0.60	0.73	0.79
	ISO-1	0.63	0.52	0.71	0.67	0.66	0.59	0.70	0.70
	ISO-2	0.63	0.53	0.70	0.69	0.70	0.61	0.77	0.76
	NQA-1	0.69	0.56	0.79	0.74	0.63	0.46	0.74	0.72
	NQA-2	0.56	0.39	0.71	0.60	0.64	0.49	0.74	0.69

**Table 3:** The results of identifying ideation subjects from HCs and depression subjects by XGB and LR are the highest of a 10-time train_test_split iteration. The data of HCs and depression subjects was split into two halves (−1 and −2) to reduce imbalance. Note that the maximum values presented in a row came from a single prediction that attained the highest AUC. ACC, accuracy; AUC, area under curve; SEN, sensitivity; SPE, specificity.

Model	Metric	5-Fold CV				Test			
		ACC	SEN	SPE	AUC	ACC	SEN	SPE	AUC
XGB	GFA-1	**1.00**	1.00	1.00	**1.00**	**0.85**	0.62	1.00	0.92
	GFA-2	0.80	0.83	0.78	0.91	**0.85**	0.75	0.92	**0.94**
	ISO-1	0.73	0.50	1.00	0.93	0.70	0.62	0.75	0.91
	ISO-2	0.67	0.83	1.00	**1.00**	0.80	0.62	0.92	0.89
	NQA-1	0.88	1.00	0.80	0.98	0.70	0.38	0.92	0.90
	NQA-2	0.88	1.00	0.50	0.88	0.80	0.75	0.83	0.75
LR	GFA-1	0.80	1.00	0.73	0.98	0.80	0.62	0.92	0.93
	GFA-2	0.80	1.00	0.73	**1.00**	0.80	0.62	0.92	0.93
	ISO-1	0.80	1.00	0.70	0.96	0.75	0.50	0.92	0.85
	ISO-2	0.87	1.00	0.82	**1.00**	0.80	0.62	0.92	0.92
	NQA-1	0.93	0.80	1.00	**1.00**	0.65	0.25	0.92	0.92
	NQA-2	0.81	0.60	0.73	0.82	0.65	0.62	0.67	0.79
